# Global drivers of herbicide‐resistant weed richness in major cereal crops worldwide

**DOI:** 10.1002/ps.6800

**Published:** 2022-02-02

**Authors:** Philip E Hulme

**Affiliations:** ^1^ Bio‐Protection Research Centre Lincoln University Canterbury New Zealand

**Keywords:** best subset regression, crop competitiveness, international herbicide‐resistant weed database, macroecology, sampling effects, sustainable intensification

## Abstract

**BACKGROUND:**

The number of herbicide‐resistant weeds differs across the globe but the reasons for this variation are poorly understood. Taking a macroecological approach, the role of six drivers of herbicide resistance in a country was examined for barley, maize, rice and wheat crops worldwide. Drivers captured agronomic measures (crop harvested area, herbicide and fertilizer input) as well as sources of sampling bias that result in under‐reporting of herbicide resistance (human population density, research intensity and time since the first record of resistance).

**RESULTS:**

Depending on the crop, best subset regression models explained between 60% and 80% of the variation in herbicide‐resistant weeds recorded in countries worldwide. Global prevalence of herbicide‐resistant weeds is likely underestimated, especially in countries with limited capability in herbicide research. Numbers of resistant weeds worldwide will continue to increase. Agricultural intensification, captured by fertilizer and herbicide input, as well as further expansion of crop harvested area are primary drivers of future herbicide‐resistant weeds.

**CONCLUSION:**

Because the evolution of herbicide resistance lags behind the selection pressures imposed by fertilizer and herbicide inputs, several countries (e.g. Brazil, South Africa, Uruguay) appear to exhibit a ‘herbicide resistance debt’ in which current agronomic conditions have set the scene for higher numbers of herbicide‐resistant weeds than currently observed. Future agricultural expansion will lead to more herbicide‐resistant weeds, especially in developing countries as their economies grow and where herbicide resistance is currently under‐reported. A global strategy for increasing national capability in herbicide resistance research is needed. © 2022 The Author. *Pest Management Science* published by John Wiley & Sons Ltd on behalf of Society of Chemical Industry.

## Graphical abstract

The drivers most frequently included to explain global variation in the number of herbicide‐resistant weeds were those related to biases in sampling effort (time since first record and research effort) suggesting global prevalence of herbicide resistance is underestimated.

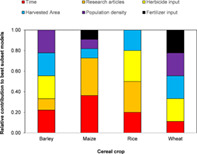

## 1 INTRODUCTION

Herbicide‐resistant weeds pose an increasingly important constraint upon sustainable agricultural practices around the world.^1^, ^2^ The risk of herbicide‐resistant weeds has limited the practise of using lower herbicide application rates^3^ and encouraged the use of more persistent and less environmentally friendly herbicides.^4^ For over half a century research has focused largely on understanding herbicide resistance at the phenotypic, physiological and genetic scales in order to develop mitigation strategies.^5^, ^6^ Nevertheless, even for a single crop, considerable variation exists in the number of herbicide‐resistant weeds recorded in different countries,^7^ suggesting that a larger scale perspective might shed new light on a long‐standing agronomic problem. Furthermore, global patterns in herbicide‐resistant weeds are changing and although historically a problem for developed countries, the increasing intensification of agriculture in developing countries has resulted in a rapid increase in cases of herbicide‐resistant weeds in these regions since 1990.^8^

In contrast to attempts to understand the drivers of herbicide resistance in individual countries,^9^, ^10^, ^11^, ^12^ it remains unclear why some countries experience greater numbers of herbicide‐resistant weeds than others. Such variation is likely to arise from differences in crop management, herbicide availability and weed composition across countries that lead herbicide resistance to evolve at different rates.^13^, ^14^ Countries differ in the extent they apply agronomic options that are known to reduce the rate at which weeds evolve herbicide resistance such as boosting crop competitiveness, employing more effective crop rotations, using herbicide mixtures, undertaking strategic tillage and applying precision weed management.^15^, ^16^ Furthermore, weed species do not all evolve resistance to the same herbicide modes of action or exhibit similar mechanisms of resistance to the same herbicide (e.g. target vs. non‐target)^17^ and thus may not respond to external drivers in a homogeneous manner. Given these management interventions and resistance mechanisms influence selection pressures on herbicide resistance at a field scale, their general impact might be difficult to discern when comparing multiple countries at a national scale across the globe. Nevertheless, macroecological perspectives have proven powerful in explaining global variation in processes that are often shaped by specific local contexts.^18^ For example, national‐scale variables have been used to explain worldwide patterns in the weed richness of cereal crops,^19^ the geographical distribution of crop pests and pathogens,^20^, ^21^ the risk of invasive conifers escaping from commercial forestry plantations in the northern and southern hemispheres,^22^ yields of cereal crops in 188 nations over 40 years,^23^ and even the economic gains in crop production from bee pollination across Europe.^24^ But what are the national‐scale variables that might be important in shaping the worldwide patterns in the number of herbicide‐resistant weeds?

At least six variables assessed at a national scale that capture agronomic practices and sampling effort are expected to influence the number of herbicide‐resistant weeds recorded in different countries (Table 1). Among the agronomic variables, herbicide input is widely understood to be important. Herbicide usage in crop production varies markedly across the globe^25^ and it might be expected that the magnitude of herbicide input per unit area of cropland would be an important predictor of the number of herbicide‐resistant weeds in a country. In addition, countries in which a particular crop has been planted extensively should have a higher number of herbicide‐resistant weeds compared with countries where the crop is not grown so widely. This is because as the harvested area of a crop increases, so too does the species richness, frequency and abundance of weed species.^26^ Because the frequency of spontaneous herbicide resistance in weeds is of the order of 10^−8^,^27^ the more abundant and diverse the weed flora the greater the probability of such a mutation occurring. Crop management may also act to reduce the selection pressure on herbicide resistance.^15^ The application of fertilizer can increase the competitiveness of the crop and thus reduce weed performance that, when integrated with appropriate timing of herbicide application, could result in a lower likelihood of weeds evolving herbicide resistance.^28^

**TABLE 1:** Description of the six explanatory variables included in the analysis of country‐level variation in the number of herbicide‐resistant weeds worldwide. For each variable, the expected association with the number of herbicide‐resistant weeds is presented and supported by a brief rationale

Variable	Association	Rationale
Fertilizer input	Negative	Greater fertilizer input should boost crop competitiveness and reduce weed performance
Herbicide input	Positive	The more herbicide used in a country the stronger the selection pressure for resistance
Crop harvested area	Positive	Greater diversity of weeds and frequency of herbicide exposure for crops grown widely
Time since first resistance	Positive	The longer the period since herbicide resistance was recorded in a country the greater the opportunity for further evolution to occur
Research articles	Positive	The greater the research intensity on herbicides in a country the more likely resistance will be detected
Population density	Positive	Higher human population densities are correlated with greater biological sampling and thus a greater likelihood of detecting herbicide resistance

Although agronomic variables may impact herbicide resistance directly, variation among countries could also reflect sampling effects. For a particular crop, it might be expected that the longer the time since herbicide resistance was first recorded in a country, the greater the likelihood that other weed species have also become resistant. This is in part because the first record may reflect a tipping point in crop management associated with increased selection pressure for herbicide resistance,^29^ but also because different weed species evolve resistance at different rates^5^ and that the first record acts as an early warning. Higher records of herbicide‐resistant weeds could also reflect greater research intensity on herbicides and resistant weeds in those countries investing more heavily in agronomic and agrochemical research and development. Similarly, botanical recording tends to be biased in areas of high population density^30^, ^31^ and this could translate to increased detectability of herbicide resistance. The identification of herbicide‐resistant weeds requires specialist expertise and facilities to undertake standard assays^32^, ^33^ and thus national capability in this discipline, measured by research intensity on herbicide resistance, may also shape the likelihood of herbicide‐resistant weeds being detected. These three factors would need to be taken into account to avoid the global patterns of herbicide‐resistant weeds simply reflecting greater sampling effort and interest in crop weeds in some countries more than in others.^19^

Although there are clear expectations as to how these explanatory variables might influence the number of herbicide‐resistant weeds recorded in different countries, there has been no attempt to tease them apart and determine whether there are consistent national‐scale drivers of herbicide resistance. Such information is crucial to identify those countries that are on a future trajectory towards increased numbers of herbicide‐resistant weeds. The results might also point to the need for national guidelines for herbicide resistance management rather than the decision being left to the individual farmer. Given this background, the overall objective of this study was to use a macroecological approach to explain global variation among countries in herbicide resistance, examine how herbicide resistance varies among different cereal crops worldwide, and to identify the relative importance of agronomic variables and sampling effort in these patterns.

## 2 MATERIALS AND METHODS

### 2.1 Data sources

Data on the total number of herbicide‐resistant weed species recorded in different countries was retrieved from the International Herbicide‐Resistant Weed Database^34^ (www.weedscience.org) on 19 June 2021. Selection of the target crops was based on each having a minimum of 15 countries where the crop was currently grown and in which at least one herbicide‐resistant weed had been recorded. As a result, analyses focused on the four globally important cereal crops: barley (*Hordeum* spp. and *H. vulgare*), maize (*Zea mays*), rice (*Oryza sativa* and *O. glaberrima*) and wheat (*Triticum aestivum*, *T. durum* and *Triticum* spp.).

Socio‐economic and agronomic explanatory variables were either extracted directly or derived from data archived in the Food and Agriculture Organisation FAOSTAT database^35^ (fao.org/faostat). The FAOSTAT database contains multiple variables describing different aspects of global agriculture including production, agrochemical inputs, trade, agri‐environment indicators, investment and macrostatistics. To avoid overfitting models, an informed, hypothesis‐led approach was adopted for variable selection. A long‐list of socio‐economic and agronomic variables was further trimmed by excluding highly correlated (*r* > 0.7) variables (e.g. input of N, P and K fertilizers; agricultural land area, crop yield, crop imports). This selection process led to a set of four explanatory variables that exhibited relatively low collinearity (Table S1): human population density (in people per km^2^), nitrogen‐based fertilizer input per area of cropland (t km^−2^), herbicide input per area of cropland (t km^−2^), and the harvested area (ha) of the specific target cereal crop. Data for each of these variables were averaged over the period 1990–2018 (coinciding with the latest data in FAOSTAT).

For each country, the earliest record of herbicide resistance in each of the four cereal crops was extracted from the International Herbicide‐Resistant Weed Database. This date was used to calculate the number of years before 2021 that herbicide resistance first appeared in order to provide a measure of the time since first record of herbicide resistance. To capture research effort on herbicides in each country, the following search term was used: ((herbicide and weed) NOT ‘herbicide resistan*’) across the titles, abstracts and keywords of articles archived in the Clarivate Analytics Web of Science over the same period as the FAOSTAT data (between 1990 and 2018). The Boolean operator excluding articles addressing herbicide resistance from the search aimed to limit the risk of circularity where more research is undertaken on herbicide–weed interactions when there are more frequent occurrences of herbicide resistance in a country. Although searching for terms in English may limit the range of publications examined, it does capture the bulk of international peer‐reviewed agronomic literature.^19^ The search term was run separately for each country picking up all publications in which at least one author was affiliated with an institution in that country. The distributions of the dependent (except for wheat) and all explanatory variables were log_10_‐transformed to reduce any heteroscedastic biases and improve the linearity of the relationships in the regression models.

### 2.2 Statistical analysis

An initial analysis of variance (ANOVA) was undertaken across all four crops to identify any differences in the mean values of the six explanatory variables (log_10_‐transformed) that might lead to variation in the trends encountered. Subsequently, separate best subset regression analyses were undertaken for each target cereal crop using only the countries where the target crop was grown and for which there was a least one record of a herbicide‐resistant weed in that crop. An information theoretic approach to model selection was used to identify the relative importance of the different explanatory variables in the overall variation in the number of herbicide‐resistant weeds found in different countries. Information theoretic model selection using Akaike’s information criterion (AIC) offers significant advantages over standard regression analysis when applied to problems of complex causality that may include nonlinear relationships and collinearity among independent variables.^36^ Owing to the relatively small sample sizes in the herbicide resistance data sets, the second‐order AIC (ΔAICc) was used as a means for model selection to rank all models.^37^ The AICc is an estimate of the out‐of‐sample prediction error and reflects the relative quality of different statistical models for a given set of data.^38^

The regression analyses considered all possible models by running every combination of the explanatory variables (for six explanatory variables this results in 64 models) and each model ranked in relation to ΔAICc.^39^ The best subset models were identified as those whose ΔAICc was within two units of the minimum AICc score across all models because these models are generally viewed as having substantial empirical support.^40^ Although this represented a large number of competing models to consider, it was a small proportion of the potential macroecological variables that could have been included in the analysis. By focusing on only six variables for which there was an *a priori* expectation (Table 1), the analyses were not a ‘shot‐gun’ attempt to find significant variables, but were more precisely testing the relative effects of a realistic set of candidate predictors.^41^

The best subset analysis enabled the relative importance of the six explanatory variables to be gauged. The selection of the most parsimonious model among the best subset targeted the model that included the fewest explanatory variables but that also showed low collinearity (variance inflation factor <2) and retained similar predictive power as more complex models as determined by the tenfold cross‐validated *R* ^2^.^42^, ^43^, ^44^ This approach was designed to prevent the frequent overfitting that limits best subset regression analysis and thus ensure that the selected models contained no irrelevant explanatory variables and were able to adequately predict future observations.^45^ Examination of the standardized residuals from each model fit was used to identify countries with a much lower number of herbicide‐resistant weeds than expected, potentially indicating a higher potential risk of future herbicide resistance. All analyses were undertaken in Minitab 20.2.^46^

## 3 RESULTS

### 3.1 Broad patterns in explanatory variables

**FIGURE 1:**
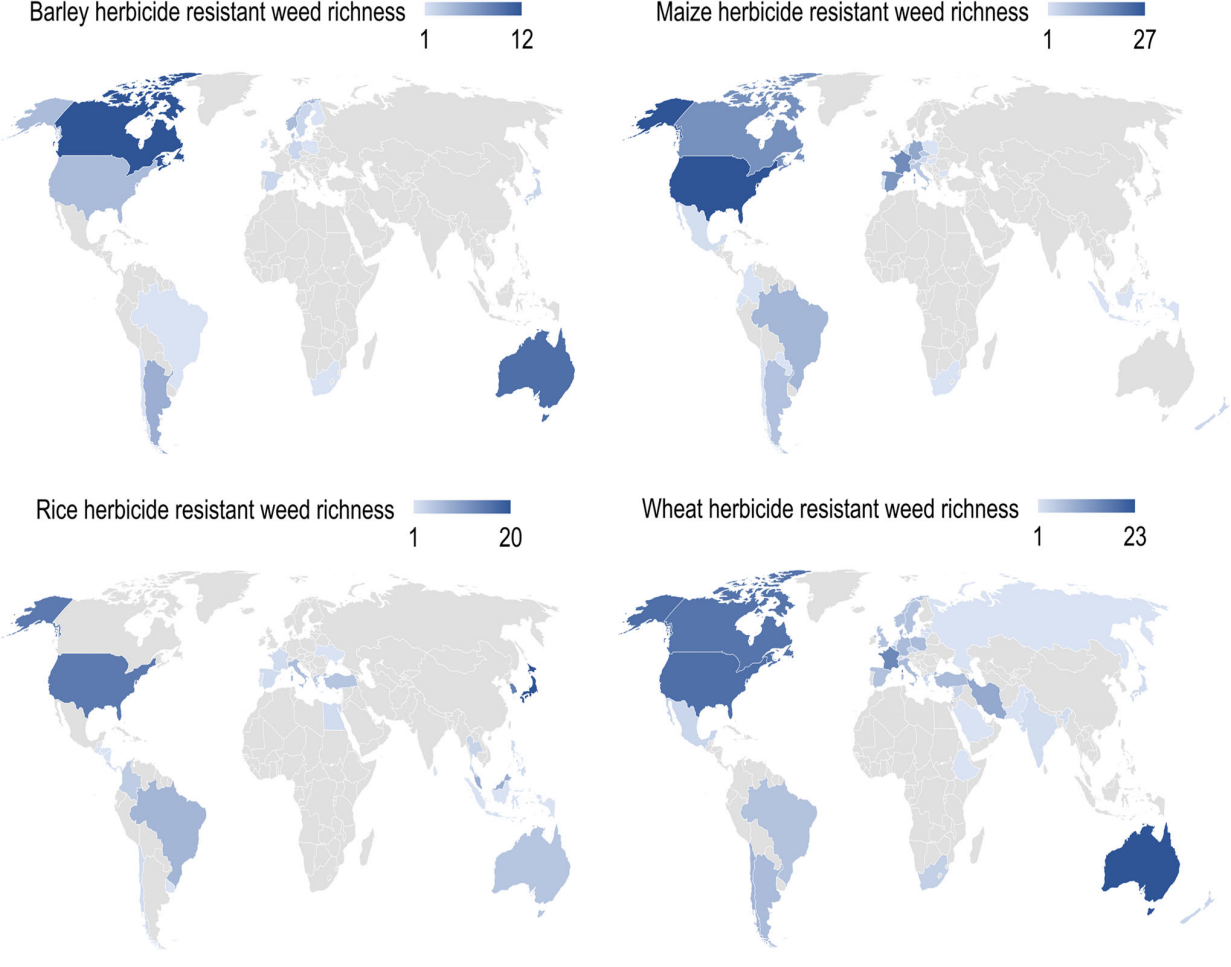
Global distribution of herbicide‐resistant weed richness in barley, maize, rice and wheat crops worldwide as retrieved from the International Herbicide‐Resistant Weed Database^34^ (www.weedscience.org) on 19 June 2021.

The average number of herbicide‐resistant weed species found per country was low ranging from 3.11 in barley to 6.14 in wheat, but the almost twofold difference among the four cereal crops was of only borderline significance (Table 2). The countries with the highest recorded number of herbicide‐resistant weeds differed according to the cereal crop examined. Thus, the highest number of herbicide‐resistant weeds in barley was recorded in Canada, whereas it was the USA for maize, Japan for rice and Australia for wheat (Figure 1). There was limited overlap in countries included in the analyses for each crop. Of a total 65 countries in the data set, only four (Brazil, Chile, Spain and USA) were included in the data for all four crops, whereas more than half of the countries (52%) were associated with just one crop. Rice was the most distinctive of all four crops with 46% of countries specific to that crop, notably several in Central America (Costa Rica, El Salvador, Guatemala, Honduras, Nicaragua and Panama). This heterogeneity in the composition of countries across all four crops led to significant differences in the mean values of several explanatory variables (Table 2). The crop harvested area of wheat was significantly higher than that of either barley or rice, whereas the number of research articles addressing herbicides was significantly lower for countries in the rice data set than for either barley or wheat. The appearance of herbicide resistance in weeds in barley was significantly more recent than in any of the other three crops. This heterogeneity in the agronomic variables and sampling effort of each crop likely promote quite different explanatory models of the number of herbicide‐resistant weed species.

**TABLE 2:** Means and standard errors for the mean number of herbicide‐resistant weeds and each explanatory variable used in the regression analyses for each cereal crop

	Barley	Maize	Rice	Wheat	*p*‐value
Number of countries	17	30	30	37	
Mean number of weeds	3.12 ± 0.79	5.07 ± 1.16	4.27 ± 0.86	6.14 ± 0.89	0.079
Population density (km^−2^)	0.75 ± 0.23	1.14 ± 0.20	1.19 ± 0.21	1.19 ± 0.20	0.265
Fertilizer input (t km^−2^)	94.65 ± 22.96	94.37 ± 11.71	79.69 ± 11.58	86.66 ± 10.67	0.652
Herbicide input (t km^−2^)	1.26 ± 0.20	1.58 ± 0.24	1.77 ± 0.31	1.15 ± 0.17	0.201
Harvested area (×10^6^ ha)	1.07 ± 0.32^b^	3.36 ± 1.41^a^	2.2 ± 1.09^b^	4.77 ± 1.26^a^	**0.008**
Research articles	461.65 ± 230.47^a^	292.57 ± 133.97^b^	250.47 ± 134.13^b^	301.95 ± 108.83^a^	**0.042**
Years since first record	17.59 ± 2.63^b^	27.63 ± 2.63^a^	22.77 ± 1.51^a^	23.46 ± 1.57^a^	**0.048**

The *p*‐value of a one‐way analysis of variance on log‐transformed data is also presented and where statistically significant variation was found across the four crops the *p*‐value is in bold and different superscripts indicate significant differences between means as assessed by Fisher’s Least Significant Difference (LSD).

### 3.2 Relative importance of explanatory variables for each crop

**FIGURE 2:**
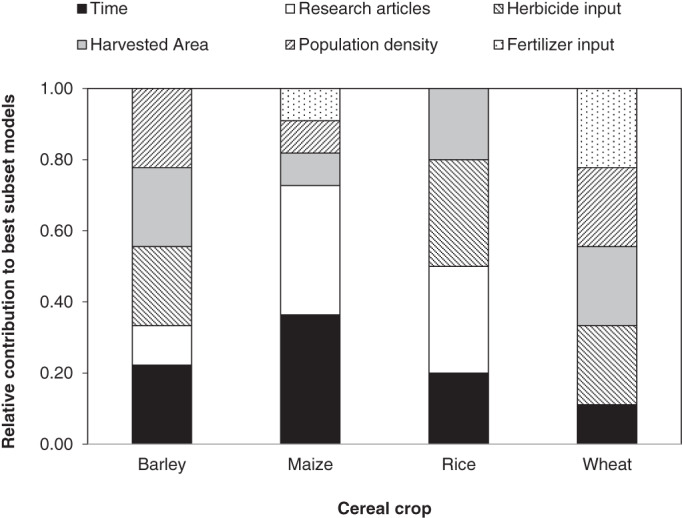
Relative contribution of six explanatory variables to best subset regression models for the number of herbicide‐resistant weeds found in countries growing barley, maize, rice and wheat. Explanatory variables include agronomic factors (crop harvested area, herbicide and fertilizer input) as well as potential sources of sampling biases (human population density, research intensity as measured as the number of research articles published on herbicides in a country, and the time since the first record of resistance).

For every crop, models retaining only a subset of the explanatory variables provided a better fit to the data in terms of adjusted and cross‐validated *R* ^2^ and measures of collinearity than a full model (Table 2). Across all crops, the most frequently included explanatory variable was the time since the first recorded instance of herbicide resistance in a country, followed by the number of research articles published that addressed herbicides (Figure 2). Herbicide input and crop harvested area were the two agronomic variables most frequently included in models. There was no occasion on which the same best subset of explanatory variables was repeated in different crop species, suggesting the suite of variables associated with the number of herbicide‐resistant weeds in a country are crop dependent. However, for individual crops, different models showed considerable homogeneity, often only differing in one explanatory variable.

The models that explained the greatest amount of variation in the number of herbicide‐resistant weed species in a country were those for barley. Over 75% of the variation in numbers of herbicide‐resistant weeds in a country could be explained by only four variables. As might be expected, the number of herbicide‐resistant weed species in a country was positively associated with the level of herbicide input, crop harvested area and the time since the first record of herbicide resistance (Table 3). However, contrary to expectations, the number of herbicide‐resistant weeds in country was negatively associated with human population density.

**TABLE 3:** Summary of the regression models describing the role different explanatory variables in the number of herbicide‐resistant weeds found in four major cereal crops worldwide

		Model goodness‐of‐fit					Explanatory variables					
Crop	Variables	*R* ^2^	*R* ^2^ (adj)	*R* ^2^ (cross)	AICc	VIF (max)	Population density	Fertilizer input	Herbicide input	Harvested area	Time	Research articles
Barley	4	81.5	75.4	66.2	4.29	1.19	−0.339		0.334	0.496	0.436	
	5	86.1	79.7	73.3	5.50	2.72	−0.310		0.463	0.723	0.560	−0.350
	Full	87.5	79.9	64.0	11.23	2.78						
Maize	2	69.6	67.2	62.7	12.98	1.13					0.286	0.679
	3	71.9	68.4	61.9	13.77	1.17		−0.162			0.341	0.687
	3	71.4	67.8	62.3	14.33	1.42				0.146	0.339	0.617
	3	71.2	67.6	62.8	14.51	1.14	−0.123				0.307	0.671
	Full	73.2	65.5	52.2	23.35	2.83						
Rice	3	63.1	58.6	53.0	12.84	1.27			0.344	0.296		0.610
	4	65.0	59.1	52.7	14.52	1.42			0.323	0.243	0.148	0.607
	3	60.8	56.1	48.8	14.57	1.03			0.356		0.219	0.683
	Full	65.0	55.4	41.2	21.88	1.56						
Wheat	4	64.9	60.4	48.3	199.03	1.63	−0.488	0.404	0.440	0.678		
	5	66.3	60.6	49.8	200.75	1.71	−0.501	0.396	0.370	0.616	0.136	
	Full	66,3	59.3	43.3	204.07	4.23						

For each crop, goodness‐of‐fit statistics are presented for each model included in the best subset as well as the standardized regression coefficients for each variable included in the model. For comparison, goodness‐of‐fit statistics are also presented for the full model that included all six explanatory variables. Goodness‐of‐fit statistics are the overall, adjusted and cross‐validated *R* ^2^, the corrected Akaike’s information criterion (AICc) and the largest variation inflation factor (VIF) recorded by an explanatory variable included in the model.

In the case of maize, approximately two‐thirds of the variation was explained in the regression models, with the number of research articles and the time since first herbicide record being the most important explanatory variables. Indeed, the most parsimonious model included only these two variables (Table 3). For rice, regression models explained just under 60% of the variation in the numbers of herbicide‐resistant weeds in a country and two variables were common to all models: herbicide input and the number of research articles. A model that included these two variables as well as crop harvested area appeared the simplest explanation of variation in herbicide‐resistant weeds in rice worldwide. Finally, for wheat, models explained approximately 60% of the variation and four explanatory variables were common to the best subsets: human population density, fertilizer input, herbicide input and wheat harvested area.

### 3.3 Testing the expectations

The regression analyses present mixed support for the expectations set out for each of the explanatory variables (Table 1). Trends consistent with the expectation of a positive relationship with the number of herbicide‐resistant weeds in a country were always found for herbicide input, crop harvested area and time since first record. There was mixed support for fertilizer input and the number of research articles being associated with a greater number of herbicide‐resistant weeds in a country, although the sign of the regression coefficients was consistent within a single crop species. There was only one occasion on which the number of research articles was negatively related to the number of weed species in a country and thus evidence against the expectation that greater research effort is related to higher detection of herbicide‐resistant weeds was weak, especially when examining only the most parsimonious models.

Fertilizer input was negatively related to the number of herbicide‐resistant weeds in a country for maize, but positively related in wheat (Table 3). The inclusion of a positive coefficient for fertilizer input in both best subset models in wheat argues against increased fertilization acting to reduce herbicide‐resistant weed species through a greater crop competitive ability. Wheat was the only crop for which fertilizer and herbicide inputs were significantly correlated with each other (*r* = 0.337, *p* = 0.045; Table S1) and thus the positive coefficient for fertilizer input might simply reflect that this also coincides with greater herbicide input. Contrary to expectations, there was a consistent negative relationship between human population density and the number of herbicide‐resistant weed species in a country. Thus, although population density is clearly an important explanatory variable of the number of herbicide‐resistant weeds in a country, it does not appear to be consistent with increased sampling effort. Human population density in a country was positively correlated with fertilizer input for all four cereal crops (Table S1) and significantly so for maize (Pearson *r* = 0.555 *p* < 0.001), rice (*r* = 0.45 *p*= 0.0012) and wheat (*r* = 0.530, *p* < 0.001). Thus, it is conceivable that population density might act as a proxy for fertilizer input.

### 3.4 Variation among countries

All best subset models for each crop had reasonable predictive power as captured by their cross‐validated *R* ^2^ values, and thus may provide some insights into future herbicide‐resistant weed risks. For each crop, the top three countries for which predictions overestimated the number of herbicide‐resistant weeds, and thus potentially indicate a higher future risk, were identified using the residuals from the fit of the most parsimonious model: barley (Brazil, South Africa and Finland), maize (Poland, South Africa and Bulgaria), rice (Uruguay, Portugal and Spain) and wheat (Russia, the Netherlands and Lithuania). Although these countries represent a wide range of climates and levels of economic development, they all have relatively few herbicide‐resistant weeds recorded to date (Figure 1).

## 4 DISCUSSION

Despite considerable differences in crop production systems around the world^47^ a relative limited suite of national‐scale variables was able to explain between 60% and 80% of the variation in numbers of herbicide‐resistant weeds recorded in countries worldwide. Although the suite of explanatory variables retained in the regression models differed among the four crops, the overall patterns suggest that global variation in the number of herbicide‐resistant weeds is as much a function of sampling effort as agronomic practice.

It is logical to expect the number of herbicide‐resistant weeds in a country to increase over time.^2^ However, many countries with few herbicide‐resistant weeds have also only recorded these weeds relatively recently, suggesting that they may be at the beginning of a future trajectory of increasing cases of herbicide resistance. In many cases, evidence of only a few records of herbicide resistance in a country may also reflect limited surveys of herbicide‐resistant weeds that would be associated with a low intensity of national research on herbicides. It is widely recognized that geographical patterns of plant species richness are influenced by sampling effort, particularly biases in botanical recording.^19^, ^48^, ^49^ Sampling biases are expected to be more severe for records of herbicide resistance because specific protocols are necessary to test for the phenomenon that require specialist herbicide knowledge and expertise.^32^, ^33^, ^50^ Taken together, these two findings highlight global patterns in herbicide‐resistant weeds are most likely underestimated, especially in countries with limited capability in herbicide research, and that numbers of herbicide‐resistant weeds worldwide will continue to increase particularly in countries with only a short history of herbicide‐resistant weeds.

The expectation that the number of herbicide‐resistant weeds in a country would be positively related to human population density was not supported. Although sampling of plant species is often biased towards regions of high population density,^49^ the means of detecting herbicide resistance are more complex than simply collecting a specimen for a herbarium collection. This specialist knowledge (and associated facilities) may not scale with population density. Indeed, for none of the four cereal crops was a significant relationship found between population density and the number of research articles addressing herbicides (Table S1). By contrast, the significant positive relationship between population density and the number of herbicide‐resistant weeds in a country may reflect increased agricultural intensification. There is both theoretical and empirical evidence that increasing rural population density will leady indirectly to greater agricultural intensification by raising the demand for modern inputs such as fertilizers, improving the flow of information, reducing transaction costs and encouraging institutions to develop and improve agricultural production.^51^, ^52^ The finding that, at a global scale, there was a positive correlation between population density and fertilizer input for all four cereal crops is consistent with its indirect role in agricultural intensification.

The harvested area of the target cereal crop was the agronomic variable most frequently associated with the number of herbicide‐resistant weeds and points to the importance of chance in the evolution of herbicide resistance. The more extensive the harvested area, the greater the range of weed species, and number of individual plants of widespread weed species, that will be exposed to herbicide application, therefore increasing the probability of a spontaneous mutation that results in herbicide resistance. Forecasts suggest that to meet increasing population demands and *per capita* income growth, the area of stable crops worldwide will likely increase by 10% by 2050.^53^ Consequently, if current herbicide application strategies remain unchanged then the number of herbicide‐resistant weeds will likely increase with the expansion of crop harvested area.

Herbicide input was a significant explanatory variable in the best subset models for all crops except maize. Herbicide application plays an obvious role in the evolution of herbicide resistance in weeds, and it was expected that the number of herbicide‐resistant weeds in a country would be positively correlated with herbicide input. In many countries, herbicide inputs have been increasing steadily over time.^25^, ^54^, ^55^ Worryingly, as the economies of developing countries grow, so labour costs are expected to rise and herbicide application will become an increasingly attractive option for farmers.^56^ This is particularly the case for rice where manual weeding is often the most popular, but time‐consuming, method of weed control.^57^

In contrast to herbicide input, fertilizer input only had a modest contribution to the best subset regression models. For maize, fertilizer input had a weak negative relationship with the number of herbicide‐resistant weeds in a country. This contrasts with wheat, where the significant relationship for fertilizer input was positive. The expectation was that fertilizer input should increase the competitiveness of the crop and result in reduced weed performance and a lower likelihood of herbicide resistance evolving. The lack of a consistent response to fertilizer input is in line with mixed evidence for the effect of nitrogen application on the competitive ability of cereal crops against weeds. The outcome of fertilization on weed–crop competition is context dependent and shaped by underlying soil fertility, the timing of crop and weed emergence, maximum heights of the weed and crop, and the relative responsiveness of weeds and crop to nitrogen addition and even the form of nitrogen (e.g. nitrate or ammonium) that is applied.^28^ Wheat was the only crop for which a significant positive correlation was found between fertilizer and herbicide input, and the positive coefficient for fertilizer input could be acting as a proxy for herbicide input.

Nevertheless, it should be borne in mind that herbicides and fertilizers are not applied to all cropland at a similar rate, nor are all herbicides used in similar amounts^58^, ^59^ and thus the total volume used in a country may over‐ or underestimate the amount applied to particular crops. For example, per unit area, an order of magnitude greater volume of herbicide is applied to maize compared with wheat crops in the USA.^60^ Although all four cereal crops were a significant component of agricultural land use in the countries where they are grown, on average they still accounted for only a moderate percentage of total cropland that would receive herbicide and fertilizer input: barley (12.4%), maize (12.1%), rice (12.6%) and wheat (18.6%). A further challenge is that there is likely to be a considerable lag between changes in agronomic conditions, such as increased herbicide input, and the recording of herbicide‐resistant weeds due both to the time required for natural selection of weed genotypes to occur and the delay between the appearance of herbicide resistance and its subsequent detection.

## 5 CONCLUSIONS

In summary, despite the evolution of herbicide resistance largely reflecting specific local management contexts,^13^, ^14^, ^61^ a global analysis generated robust and sensible insights into the drivers of herbicide resistance for individual countries. Furthermore, the results are of considerable concern. It is highly likely that the global picture of herbicide‐resistant weeds currently underestimates the significance of the problem with a considerable risk of under‐sampling occurring in countries with limited relevant research capability. Many of these countries have only detected herbicide resistance relatively recently and the evidence points to a trajectory of increasing numbers of herbicide‐resistant weeds in the future. Because of time lags between changes in selection pressures on weeds for herbicide resistance and the subsequent detection of herbicide‐resistant weeds, many countries already face a ‘herbicide resistance debt’ that has set the scene for future herbicide‐resistant weeds. Such trends would be sufficiently worrying on their own, but the important role that crop harvested area and measures of agricultural intensification (fertilizer and herbicide inputs, human population density) suggests that the pressure to increase agricultural productivity, particularly in developing countries, will come at a cost of increased numbers of herbicide‐resistant weeds. A global strategy for increasing national capability in the detection and management of herbicide‐resistant weeds, particularly in those countries with limited investment in agricultural extension, should be a priority.

## CONFLICTS OF INTEREST

The author declares that he has no known competing financial interests or personal relationships that could have appeared to influence the work reported in this paper.

## Supporting information


**Table S1.** Pearson correlation coefficients between explanatory variables (log_10_ transformed) for each of four cereal crops. Values in bold are significant at the *p* < 0.01 level, while those in italics are significant at the *p* < 0.05 level.

## Data Availability

The data that support the findings of this study are available in International Herbicide‐Resistant Weed Database at www.weedscience.org and the ‐ Food and Agriculture Organisation FAOSTAT database at fao.org/faostat.
