# Supplementary material for: Global drivers of herbicide‐resistant weed richness in major cereal crops worldwide
Source: Pest Manag Sci. 2022 Feb 2;78(5):1824–32. doi: 10.1002/ps.6800 (PMC9306702; doi:10.1002/ps.6800)
Supplement: Supplementary file 1 — Table S1. Pearson correlation coefficients between explanatory variables (log10 transformed) for each of four cereal crops. Values in bold are significant at the p < 0.01 level, while those in italics are significant at the p < 0.05 level. [file PS-78-1824-s001.docx]

**Table S1.** Pearson correlation coefficients between explanatory variables (log_10_ transformed) for each of four cereal crops. Values in bold are significant at the P < 0.01 level, while those in italics are significant at the P < 0.05 level.

| *Barley* | Population density | Fertilizer input | Herbicide input | Harvested Area | Yield | Research articles |
| --- | --- | --- | --- | --- | --- | --- |
| Fertilizer input | 0.418 | 1.000 |  |  |  |  |
| Herbicide input | 0.116 | -0.018 | 1.000 |  |  |  |
| Harvested Area | -0.245 | -0.223 | -0.184 | 1.000 |  |  |
| Research articles | -0.154 | -0.368 | 0.196 | **0.651** | -0.061 | 1.000 |
| First record year | 0.454 | 0.358 | 0.126 | -0.386 | 0.176 | -0.447 |
| *Maize* |  |  |  |  |  |  |
| Fertilizer input | **0.555** | 1.000 |  |  |  |  |
| Herbicide input | -0.061 | 0.191 | 1.000 |  |  |  |
| Harvested Area | *-0.365* | **-0.478** | -0.323 | 1.000 |  |  |
| Research articles | 0.024 | 0.220 | 0.097 | 0.316 | **0.465** | 1.000 |
| First record year | -0.102 | -0.285 | -0.217 | 0.236 | *-0.461* | *-0.422* |
| *Rice* |  |  |  |  |  |  |
| Fertilizer input | *0.45* | 1.00 |  |  |  |  |
| Herbicide input | -0.12 | 0.06 | 1.00 |  |  |  |
| Harvested Area | 0.19 | 0.21 | -0.28 | 1.00 |  |  |
| Research articles | 0.00 | *0.39* | -0.06 | *0.42* | **0.59** | 1.00 |
| First record year | -0.18 | -0.18 | -0.07 | -0.35 | -0.06 | -0.23 |
| *Wheat* |  |  |  |  |  |  |
| Fertilizer input | **0.530** | 1.000 |  |  |  |  |
| Herbicide input | 0.020 | *0.337* | 1.000 |  |  |  |
| Harvested Area | -0.100 | -0.206 | *-0.413* | 1.000 |  |  |
| Research articles | 0.162 | **0.485** | 0.295 | **0.480** | 0.122 | 1.000 |
| First record year | -0.095 | -0.238 | -0.312 | -0.275 | -0.058 | **-0.528** |
